# Intestinal Parasitic Infections and Associated Behavioral Risk Factors in a Squatter Community in Butwal, Nepal: A Pilot Study

**DOI:** 10.1002/hsr2.70473

**Published:** 2025-02-16

**Authors:** Shristi Bhandari, Rajendra Prasad Parajuli, Pitambar Dhakal, Kishor Pandey

**Affiliations:** ^1^ Central Department of Zoology Tribhuvan University Kathmandu Nepal; ^2^ Herbert Wertheim School of Public Health and Human Longevity Science University of California San Diego (UCSD) San Diego California USA

**Keywords:** hygiene behaviors, intestinal parasitic infections, prevalence, risk factors, squatter community, urban health and sanitation

## Abstract

**Background:**

Intestinal parasitic infections (IPIs) are among the most prevalent illnesses worldwide. The morbidity associated with IPIs results in health and economic burdens for billions of people worldwide, especially in low‐income nations. Increasing urbanization in Nepal has resulted in the formation of squatter communities in urban centers. These communities often face a disproportionate burden of health issues due to the quality of their living environment. This study aims to determine the prevalence of and risk factors for IPIs in the squatter community of Butwal, Nepal.

**Methods:**

A total of 170 individuals were recruited for a cross‐sectional study via convenience sampling. Information pertaining to demographic, socioeconomic, and behavioral factors was obtained via structured questionnaires, personal interviews, and direct witnessing during the field visit. Stool samples (*n* = 170) were examined for intestinal parasite eggs and oocysts via direct wet mount and concentration techniques.

**Results:**

Overall, the prevalence of parasites was 28.8% (5.3% protozoa, 24.7% helminths). Six species of intestinal parasites were detected. *Ascaris lumbricoides* (21.2%) was the most dominant helminthic parasite, followed by *Trichuris trichiura* (2.9%), *Entamoeba histolytica* (2.4%), *Cryptosporidium* sp. (1.8%), *Giardia lamblia* (1.8%), and hookworm (1.8%). Multivariate regression analysis revealed that participants who did not trim their nails once a week were more prone to getting a parasitic infection.

**Conclusion:**

The relatively high prevalence of IPIs among participating residents of the Butwal slum may contribute to an increased risk of IPI transmission due to inadequate hygiene behaviors. Among the participants in this study, more than one quarter stated that they do not trim their nails every week, and approximately one‐fifth indicated open defecation. Standardized health education regarding the benefits of hand and nail hygiene behaviors may contribute positively in reducing the parasitic burden and interrupting the chain of transmission in developing urban areas such as Butwal.

## 1 Background

Parasitic infections are a serious global health burden, as ~3.5 billion people are infected with intestinal parasites, the majority being children [1]. Every year, intestinal parasites contribute to 200,000 deaths worldwide [2]. Squatters (referred to as *Sukumbasi* in the Nepali vernacular language) are people who unlawfully occupy uninhabited buildings or vacant land and reside there without legal claim to the property. Communities made up of squatter residences are referred to as “slums.” Slum residents are a socioeconomically underprivileged group. They reside in poor‐quality dwellings and often work in low‐paying, hazardous job settings. These communities lack the infrastructure for waste disposal and sanitary facilities. The housing and health needs of these communities are often neglected by government policy, contributing to an overall unhealthy environment [3].

Over 1 billion individuals live in slum areas, the majority of which are in developing countries, where slum dwellers make up more than 40% of the urban population [4]. The number is rising as the pace of urbanization (urbanization rate of more than 25%) [5, 6] increases and is projected to continue rising unless local and national governments, health organizations, and the global community are able to coordinate and implement effective interventions [7]. The most often cited risk factors for IPIs are behavior, lifestyle, and dietary habits, financial status, limited access to clean drinking water and sanitation, and personal hygiene practices [8]. In developing countries, parasitic infection remains a significant health concern, particularly in resource‐limited settings. *Ascaris lumbricoides* (*A. lumbricoides*), *Trichuris trichiura* (*T. trichiura*), hookworms, *Hymenolepis nana*, and *Giardia lamblia* (*G. lamblia*) are the most common helminthic and protozoan intestinal parasites, impacting about 1 billion individuals globally [9]. It is estimated that more than 3 billion individuals are infected globally, especially children, because of their exploratory habits and poorly developed immune system [10]. Poor sanitation and a lack of safe and clean housing infrastructure provide a variety of pathways for IPI transmission in slum settlements [11].

Earlier studies conducted in southern Delhi [12], Naya Bazar, Kaski, Nepal [13], and Dhaka, Bangladesh [14] reported a high prevalence of intestinal parasites in slum areas. The most common parasites identified were *Entamoeba histolytica* (*E. histolytica*), *G. lamblia*, and *A. lumbricoides*. This finding is consistent with findings in other South Asian nations, including Nepal and Bangladesh, where *E. histolytica* is also a common intestinal protozoan parasite [15, 16]. Nepal's national deworming campaign has provided antiparasitic treatment to individuals under 18 years of age since 2004 without requiring diagnostic testing, thereby promoting widespread access and preventive measures in school‐age populations [17]. Despite mass deworming and health education programs implemented at the local level, IPIs remain the predominant cause of diarrheal illness in Nepal [11]. The prevalence of IPIs has decreased since the implementation of a 2003 deworming campaign in Nepal in the general population (i.e., broader urban and rural residents) [18, 19], in certain ethnic groups [20], and in some squatter communities [21]. However, the prevalence of IPIs remains higher among ethnic [22, 23] and squatter communities [11, 13, 24] than among the general population.

The high burden of IPIs among squatter communities may maintain a reservoir of IPIs, which poses a continuous risk of transmission. Sanitation in these communities is compromised by rapid urbanization [25], which has outpaced housing infrastructure development and insufficient public funding from the Nepali government. The lack of standardized health and hygiene education in public schools exacerbates this issue. The point prevalence of IPIs in slum populations has been widely reported in Nepal [11, 26] and in Bangladesh, Iran, and other countries [14, 27, 28]. However, comprehensive studies that emphasize potential risk factors for IPIs, such as behaviors [29, 30], and lifestyle factors [7, 10, 30], are scarce in Nepal, especially among underprivileged and vulnerable populations, such as squatter communities. This study aims to investigate the prevalence of IPIs and their associations with contextual factors in the squatter communities of Butwal, Nepal.

## 2 Methods

### 2.1 Study Sites and Study Population

**Figure 1:**
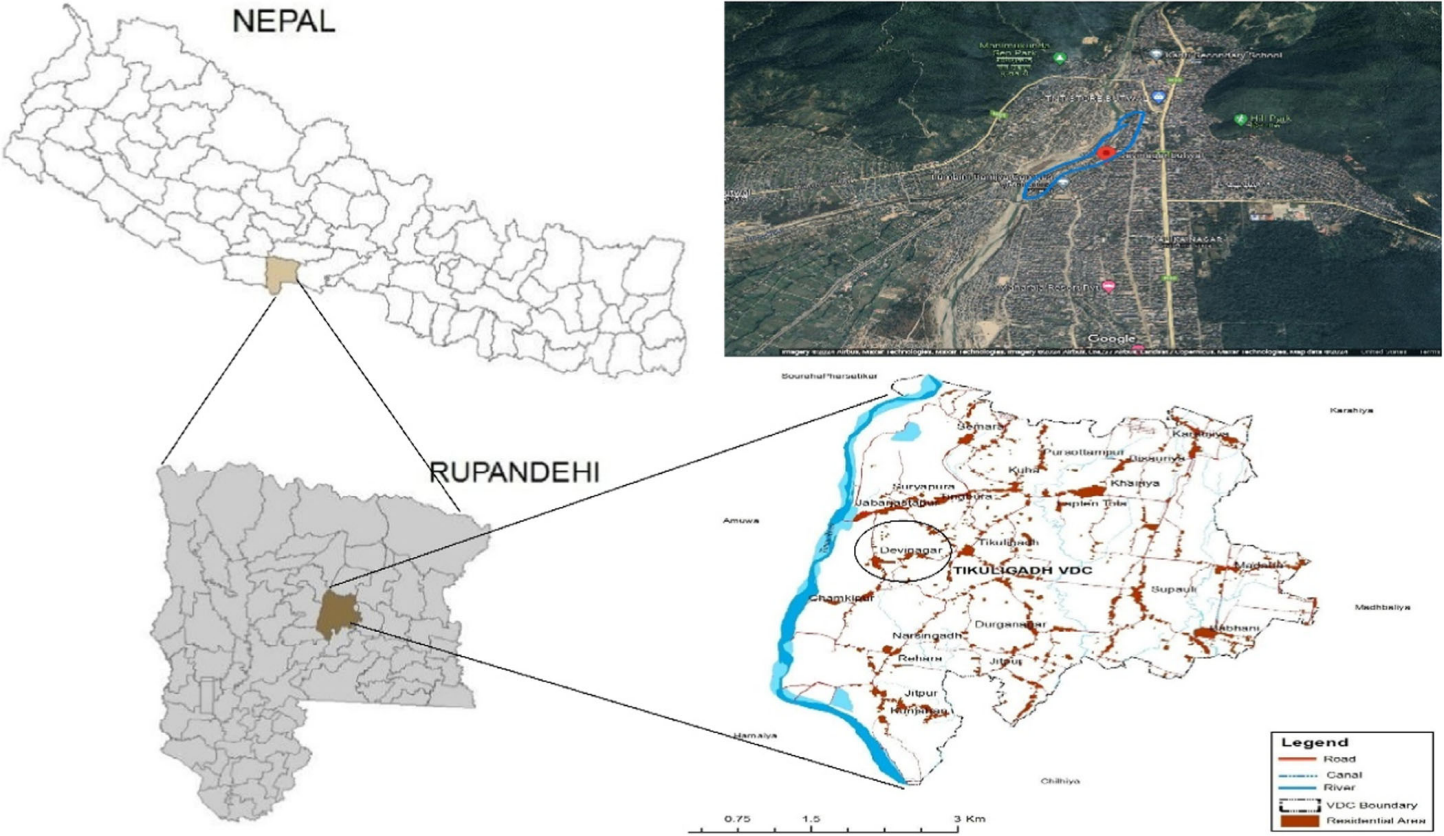
Location map of the sampling area.

This cross‐sectional study was carried out in the squatter community in Devinagar, Butwal submetropolitan city, Rupandehi district, Nepal. Butwal, a recently urbanized area, is juxtaposed with the bank of the Tinau River and lies on the northern border of the Terai plain within the Siwalik range, extending within 27.6866° N, 83.4323° E. Butwal encompasses an area of 101.61 km^2^ in Lumbini Province, located 260 km from Kathmandu, and is undergoing rapid urbanization similar to other major cities in Nepal [5, 6]. There are eight squatter communities along riverbanks, and Devinagar is home to the two clusters of squatter communities included in this study (Figure 1).

### 2.2 Sample Collection

Between October 2022 and December 2023, a total of 170 individuals were approached and recruited upon their consent in the study via a convenience sampling approach. Since the national deworming campaign commenced in 2004, which focused on younger populations [17], people younger than 18 years of age were excluded. Participants were eligible if they had not taken anthelmintics in the previous month and had resided in the study area for at least 6 months.

The objectives and procedures of the study were explained to the participants, and they were requested to sign an informed consent form if they agreed to participate in the study. Once the participants signed the consent form, they were provided a clean wooden spatula to collect approximately a thumb‐sized portion from the first part, middle part, and final part of their first stool of the morning if it was solid or semisolid and a small portion if it was watery. Each participant was requested to collect the sample in a sterile collection vial. The participants were instructed to ensure that the feces were not contaminated with urine or soil. The fecal samples were preserved in 2.5% potassium dichromate solution and stored at 4°C to preserve the parasites until laboratory analysis. A structured questionnaire survey was administered to the participants to obtain data regarding their socioeconomic, sociodemographic, behavioral, and lifestyle characteristics. Ethical approval for this study was obtained from the Institutional Review Board (IRB) of the Institute of Science and Technology (IOST), Tribhuvan University (Approval No. 23‐0061).

### 2.3 Laboratory Procedures

The preserved fecal samples were macroscopically inspected for consistency and to determine whether any adult worms were present. Direct wet mount [31], the floatation technique [32, 33], and the sedimentation technique [34] were used to detect the presence of intestinal parasites. To view the protozoan and helminth parasites, Gram's iodine stain was used to prepare the fecal smear. Gram's iodine stain is used to enhance the visibility of protozoan cysts by staining their internal structures, which aids in identifying and differentiating intestinal parasites. This stain forms a complex with the cell components, allowing for improved contrast under light microscopy. Photographs of the egg and larval stages of parasites were taken at 400x magnification under an Olympus compound microscope (BH‐2, Olympus Corporation) and identified on the basis of their morphometric characteristics (shape, size, color, cyst wall, shell structure, internal content of the cyst or oocysts or eggs or larvae) [32, 35, 36].

### 2.4 Data Processing and Analysis

The findings are presented in tables and include means, standard deviations, frequencies, and percentages. Differences between the groups were compared via *χ* ^2^ tests or Fisher's exact tests, and the mean values were compared via independent *t*‐tests. Bivariate and multivariate logistic regression analyses were conducted to evaluate the associations between IPIs and various socioeconomic, sociodemographic, and behavioral/lifestyle characteristics. In the bivariate logistic regression, each pair of variables was considered individually to assess unadjusted associations. For the multivariate model, all variables from the bivariate analysis were included to determine mutually adjusted effects. Odds ratios (ORs) and adjusted ORs (AORs) are provided with 95% confidence intervals (CIs), and a *p* value of < 0.05 was considered statistically significant. Statistical analyses were performed via SPSS statistical software.

## 3 Results

The study participants were mostly middle aged (mean age of 42 years), with a greater number of older male participants than female participants (Table 1). Compared with male participants, female participants reported better self‐rated health. Sex differences in all parameters, including age, self‐rated health status and socioeconomic status (SES), were not statistically significant between males and females. However, a greater rate of illiteracy was observed in female participants than in male participants, whereas males self‐reported poorer socioeconomic status (SES) than female participants. However, 18% of the participants reported that they did not have a closed type of toilet at home and were using an open type of toilet.

**Table 1:** Demographic, health, and socioeconomic characteristics of the study participants (*n* = 170).

	Male (*n* = 84)	Female (*n* = 86)		Total
Characteristics	Mean (SD)/*n* (%)	Mean (SD)/*n* (%)	*p* value	Mean (SD)/*n* (%)
**Sociodemographic characteristics**				
Age (in years)	43.52 (15.37)	40.26 (13.76)	NS^a^	41.87 (14.63)
Self‐rated health (1–5 maximum)	3.73 (0.63)	3.81 (0.68)	NS^a^	3.77 (0.65)
Age category				
Below 50	54 (64)	63 (73)	NS^b^	117 (69)
Above 50	30 (36)	23 (27)		53 (31)
**Socioeconomic (SES) characteristics**				
Can you read and write?				
Yes	42 (50)	40 (46.5)	NS^b^	82 (48)
No	42 (50)	46 (53.5)		88 (52)
Self‐reported SES				
Weak	45 (54)	48 (56)	NS	93 (55)
Very weak	39 (46)	38 (44)		77 (45)
Do you rear pets in the house?				
No	69 (82)	71 (83)	NS^b^	140 (82)
Yes	15 (18)	15 (17)		30 (18)
Do you rear smallholder livestock?				
No	61 (73)	65 (76)	NS^b^	126 (74)
Yes	23 (27)	21 (24)		44 (26)
Do you have toilets at your home?				
Yes, I have a closed‐type toilet	66 (79)	73 (85)	NS^b^	139 (82)
No, I have an open‐type toilet	18 (21)	13 (15)		31 (18)

^a^ Independent *T*‐test.

^b^ *Χ* ^2^ test.

**Figure 2:**
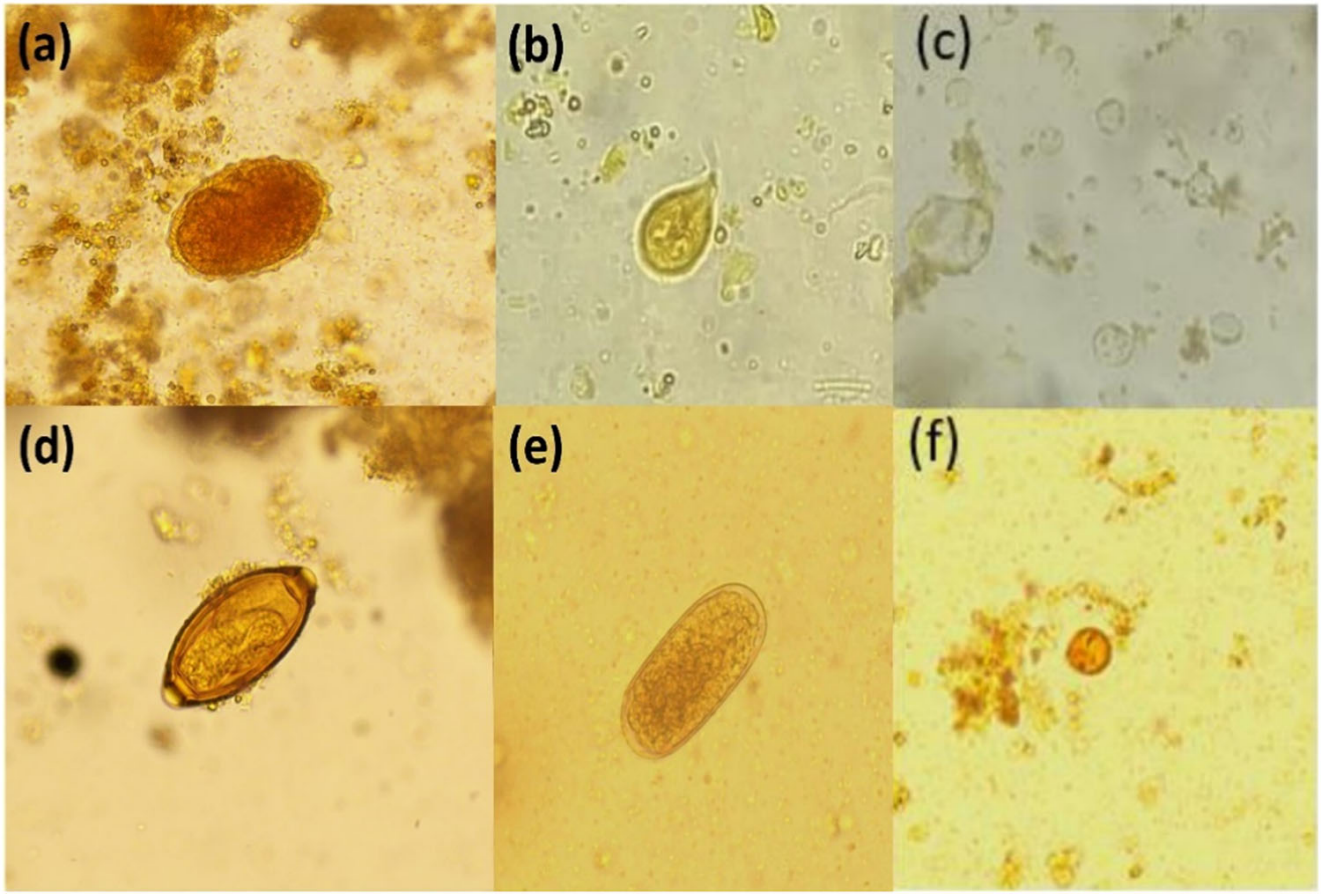
(a) Egg of *Ascaris lumbricoides* (89.66 × 53.12 μm). (b) Trophozoite of *Giardia lamblia* (25.07 × 13.16 μm). (c) Cyst of *Entamoeba histolytica* (10.45 × 8.71 μm). (d) Egg of *Trichuris trichiura* (53.41 × 21.63 μm). (e) Egg of hookworm (72 × 37 µm). (f) Oocyst of *Cryptosporidium* sp. (4 × 4 μm).

More than a quarter of the collected samples [49 out of 170 (29%)] were positive for one or more species of IPIs (Table 2). Among the six identified species of parasites, three (*E. histolytica*, *G. lamblia*, and *Cryptosporidium sp*.) are protozoa, and three (*A*. *lumbricoides*, *T. trichiura*, and hookworms) are helminths (Figure 2). Overall, *A. lumbricoides* had the highest prevalence (21.2%), followed by *T. trichiura* (2.9%) and hookworms (1.2%). Compared with male participants, female participants presented a significantly higher *T. trichiura* prevalence (Fisher's exact test *p* < 0.03) (Table 2). Overall, 24.7% of the samples presented helminth parasites, whereas 5.3% of the samples presented protozoan parasites. Three individuals, one female and two males, had duplet infections. Except for *T. trichiura*, none of the detected parasites were significantly different between males and females.

**Table 2:** Prevalence of IPIs in the Butwal squatter community (*n* = 170).

Parasite species	Male (%)	Female (%)	*Χ* ^2^ *p* value	Total *n* (%)
Protozoan parasites				
*Entamoeba histolytica*	2 (2.4)	2 (2.3)	0.68^b^	4 (2.4)
*Giardia lamblia*	1 (1.2)	1 (1.2)	0.75^b^	2 (1.2)
*Cryptosporidium* sp.	1 (1.2)	2 (2.3)	0.51^b^	3 (1.8)
Helminth parasites				
*Ascaris lumbricoides*	18 (21.4)	18 (20.9)	0.94^a^	36 (21.2)
***Trichuris trichiura***	**0 (0)**	**5 (5.8)**	**0.03** b	**5 (2.9)**
Hookworm	2 (2.4)	0 (0)	0.24^b^	2 (1.2)
Total infection	22 (26.2)	27 (31.4)	0.45^a^	49 (28.8)
Total protozoan infection	4 (4.8)	5 (5.8)	0.52^b^	9 (5.3)
Total helminth infection	19 (22.6)	23 (26.7)	0.53^a^	42 (24.7)
Duplet infection	2 (2.4)	1 (1.2)	0.49^b^	3 (1.8)

*Note:* Bold values indicate significant.

^a^ *Χ* ^2^ test.

^b^ Fisher's exact test where the cell has a count < 5.

Table 3 shows the behavioral and lifestyle characteristics of the study participants. There was no statistically significant difference in any behavioral or lifestyle characteristics between male and female participants. Most of the participants reported a nonvegetarian diet, and most of them reported that they did not bite their nails and used soap for hand washing. Most of the participants also reported having some knowledge about helminths, and that they covered food after cooking. However, about one out of three participants reported that they did not trim their nails once a week, about half of the participants did not wash green vegetables and fruits before eating, and approximately one quarter of the participants reported consuming raw meat. Approximately 25% of the participants reported walking barefoot outdoors (Figure S1).

**Table 3:** Behavioral and lifestyle characteristics of study participants (*n* = 170).

Characteristics	Male (*n* = 84)	Female (*n* = 86)	*p* value	Total
Behavioral and lifestyle characteristics	*n* (%)	*n* (%)		*n* (%)
Do you wash green vegetables and fruits?				
Yes	43 (51)	46 (54)	NS^a^	89 (52)
No	41 (49)	40 (46)		81 (48)
Are you a vegetarian?				
Yes	1 (1)	4 (5)	NS^b^	5 (3)
No	83 (99)	82 (95)		165 (97)
Do you consume raw/uncooked meat?				
No	19 (23)	29 (34)	NS^a^	48 (28)
Yes	65 (77)	57 (66)		122 (72)
Do you walk barefoot while outdoors?				
No	15 (18)	13 (16)	NS^a^	28 (17)
Yes	69 (82)	73 (84)		142 (83)
Did you trim your nails regularly every week?				
Yes	61 (73)	58 (67)	NS^a^	119 (70)
No	23 (27)	28 (33)		51 (30)
Do you cover food after cooking/eating?				
Yes	62 (74)	70 (81)	NS^a^	132 (78)
No	22 (26)	16 (19)		38 (22)
Did you deworm within 6 months?				
Yes	3 (4)	4 (5)	NS^b^	7 (4)
No	81 (96)	82 (95)		163 (96)

^a^ *Χ* ^2^ test.

^b^ Fisher's exact test while any cell has a count < 5.

The presence of any parasitic infection was higher among participants who did not trim their fingernails at least once a week than among participants who reported that they did so once a month, as indicated by the adjusted odd ratio (AOR) [2.78 95% CI (1.25–6.19)]. This study also revealed that IPIs were more prevalent among people who used open‐type toilets than among those who used enclosed‐type toilets (Table 4). However, the significance of these associations was borderline in both the univariate and adjusted multivariate models. The prevalence of IPIs was not associated with any demographic, SES, lifestyle, or other behavioral characteristics under evaluation in this study.

**Table 4:** Prevalence and odds ratios of IPIs for behavioral characteristics according to logistic regression analysis (*n* = 170).

	Any IPIs (*n* = 170)		
	Univariate		Multivariate^a^
	%	OR (95% CI)	AOR (95% CI)
**Sociodemographic (SES) characteristics**			
Age category			
Above 50	30.2	Ref	Ref
Below 50	28.2	0.91 (0.45–1.85)	0.99 (0.42–2.30)
Gender?			
Male	26.2	Ref	Ref
Female	31.4	1.29 (0.66–2.51)	1.23 (0.59–2.57)
**Socioeconomic (SES) characteristics**			
Can you read and write?			
Yes (Literate)	28.0	Ref	Ref
No (Illiterate)	29.5	1.08 (0.55–2.09)	1.25 (0.54–2.87)
Reported SES			
Weak	28.0	Ref	Ref
Very weak	29.9	1.10 (0.56–2.14)	0.72 (0.31–1.66)
Do you rear livestock?			
No	27.8	Ref	Ref
Yes	31.8	1.21 (0.58–2.56)	1.37 (0.61–3.06)
What type of toilet do you own?			
Closed	**25.9**	Ref	Ref
Open	**41.9**	2.07 (0.92–4.64)	2.29 (0.89–5.92)
**Behavioral and lifestyle characteristics**			
Do you wash green vegetable items?			
Yes	25.8	Ref	Ref
No	32.1	1.35 (0.70–2.64)	0.99 (0.46–2.14)
Are you vegetarian?			
Yes	40.0	Ref	Ref
No	28.5	0.60 (0.10–3.70)	0.33 (0.04–2.49)
Do you eat uncooked meat?			
No	25.0	Ref	Ref
Yes	30.3	1.31 (0.61–2.79)	1.28 (0.53–3.13)
Do you use soap for hand washing?			
Yes	50.0	Ref	
No	28.3	0.40 (0.05–2.89)	
Do you walk barefoot while outdoors?			
No	21.4	Ref	Ref
Yes	30.3	1.59 (0.60–4.21)	1.38 (0.46–4.16)
Did you trim your nails regularly?			
Almost every week	**22.7**	**Ref**	**Ref**
Once a month	**43.1**	**2.59 (1.28–5.21)**	**2.78 (1.25–6.19)**
Took anthelminthic within last 6 months?			
Yes	28.8	Ref	Ref
No	28.6	1.01 (0.19–5.40)	0.82 (0.13–5.22)

*Note:* Bold values indicate significant.

Abbreviations: AOR, adjusted odds ratio; 95% CI, 95% confidence interval; OR, odds ratio; %, prevalence percentage; Ref, reference.

^a^ Adjusted for variables in bivariate analysis.

## 4 Discussion

This study evaluated the prevalence of IPIs in 170 adult study participants who reside in a squatter community in Butwal, Nepal. The overall prevalence of IPIs in our study (28.8%) is comparable with the prevalence of IPIs reported in other studies. In previous research, a 27.1% prevalence was reported among slum residents of Kathmandu, Nepal [11], a 24.1% prevalence was reported among slum dwellers of Pokhara, Kaski, Nepal [13], a 25.88% prevalence was reported among slum residents of southern Delhi, India [12], and a 25% prevalence was reported in residents of urban slums in Brazil [37]. In some studies, a lower prevalence of IPIs has been reported, for example, 15.2% among slum residents of Cameroon [38], and 20.2% among slum participants of Belo Horizonte, Brazil [39]. Many earlier investigations of squatter communities, however, reported a higher prevalence of IPIs than observed in this study. For example, Chongbang and colleagues reported a 48% prevalence of IPIs in squatter communities in Dharan, Nepal [26]. This variation in the prevalence of IPIs might be attributed to varying levels of public awareness of prevention measures and changing climatic conditions [23]. Despite the ongoing national routine deworming program in Nepal, the relatively high prevalence of IPIs among our study participants may be partially explained by the poor sanitation conditions observed and reported in the participating squatter communities and inadequate personal hygiene behaviors. For example, one out of three participants reported that they did not trim their nails on a regular basis, and about half of the participants reported that they did not wash green vegetables and fruits before consumption. A quarter of the participants also reported the consumption of raw meat without cooking. In addition, more than three quarters of the participants reported that they walked barefoot outdoors. However, the majority of participants reported engaging in several healthy hygiene behaviors, including handwashing before eating, having at least basic knowledge of IPIs, and covering food, which could introduce response bias. Hence, further studies including a large number of respondents need to be conducted to confirm these findings.

The overall prevalence of all IPIs and each individual parasite was similar among males and females, even though females were found to have a higher prevalence of *T. trichiura* infections than males. Several previous investigations of IPI prevalence in South Asia have shown comparable results, with higher *T. trichiura* prevalence in females than in males [40]. However, few studies have reported a similar risk of *T. trichiura* between males and females in squatter communities [41]. In addition, a study by Zeleke and colleagues [42] reported a low prevalence of *T. trichiura* among females. This could be ascribed to geographical differences or differences in the socioeconomic characteristics and sanitary status of the study subjects [37, 40]. Although no statistically significant difference was observed between males and females in terms of the surveyed behavioral and lifestyle characteristics included in this study, more women than men reported not trimming their fingernails regularly, which may have contributed to the discrepancy in *T. trichiura* infection by sex. Although female participants demonstrated a higher prevalence of *T. trichiura* infection, their higher self‐rated health scores suggest that trichuriasis may not significantly impact their perceived health or may reflect an optimistic self‐assessment.

This study also revealed a higher prevalence of IPIs among participants who did not own closed toilets; however, this association did not reach statistical significance. Our findings are congruent with those of previous studies by Shobha and colleagues who reported a significant association between IPIs and close toilet ownership [21]. The variables selected in our study were proxy measures of socioeconomic status, sanitation, and hygiene in general. Future research in this population can gather more exact data on income, available infrastructure, and eggs per gram (epg) to strengthen the power of the study and any associations between sociodemographic and behavioral factors and IPIs. Furthermore, the odds ratios were consistently greater for both the univariate and multivariate models after adjustment. Previous studies have suggested that the prevalence of IPIs in a given community is largely determined by local ecological conditions, such as proximity to a river, warm subtropical or tropical weather, humidity, and precipitation, as well as the household microenvironment, like the existence of open defecation or sewage around houses, which may heighten exposure to infection risks among socioeconomically disadvantaged populations [43]. Since the participants were from squatter communities, poor socioeconomic status, sanitation and hygiene in general might be crucial among vulnerable populations.

This study considered a vulnerable population living near a densely populated, rapidly urbanizing area. Owing to the small study population and the sensitive nature of discussing personal sanitation issues, this study has several limitations that should be considered. The major methodological limitation of this study is the cross‐sectional sample assessment, which is unable to fully capture day‐to‐day and within‐stool variations in intestinal parasite sheds. These methods are also unable to fully capture epg data and thus limit the ability to use these results to fully measure the effectiveness of the Nepali national deworming campaign. Although the prevalence of each type of IPI in this population provides valuable information, which is useful in recommending and designing interventions to prevent the transmission of these specific intestinal parasites, the addition of intensity data would provide greater insight into the parasitic burden in individuals, families, and each participant community. Second, the smaller sample size available in the subgroup analysis may increase the risk of type II error. Finally, the possible effects of sampling bias caused by nonrandom selection of participants via convenience sampling and of response bias of participants who may want to provide the most “socially acceptable” answer to a sensitive personal hygiene question may limit the generalizability of the findings. Further studies of sanitation in this population, incorporating a larger sample size and including longitudinal studies, will help to confirm the findings of this study and provide a fuller picture of the sanitation and health needs of this population. Future research is warranted to validate the associations between hygiene behaviors and IPIs in understudied and marginalized squatter populations in Butwal, other expanding cities within Nepal, and within the greater South Asia region. Due to the morphological similarity of hookworm eggs, our study uses the term “hookworms” rather than species‐specific names, as identifying *Ancylostoma duodenale* and *Necator americanus* requires molecular methods [44, 45]. Future studies could integrate molecular techniques to distinguish between species more accurately and enhance our understanding of their prevalence and impact in this population. The findings of such research could direct public health interventions and government policy to maintain a healthy population in rapidly developing urban centers.

## 5 Conclusion

This study is one of the first to examine the prevalence of IPIs in the squatter community of Butwal, Nepal. The occurrence of helminthic and protozoan IPIs among 29% of participants from the vulnerable population of the Butwal squatter communities poses a serious public health burden in Nepal, where rapid urbanization continues and where public support for these communities is severely limited. The female participants in this study were significantly more likely than the male participants were to be infected with *T. trichiura*. Multivariate logistic regression analysis inferred that infection with at least one IPI was attributable to individual differences in personal hygiene behaviors such as nail‐trimming habits. The national implementation of a comprehensive and standardized health education program including topics such as how to properly perform fundamental sanitation and hygiene behaviors may contribute significantly to reducing the parasitic burden in developing urban areas where residents of squatter communities with limited infrastructure and social support are at increased risk of parasitic infections and disease transmission.

## Author Contributions

**Shristi Bhandari:** conceptualization, methodology, investigation, project administration, writing – original draft. **Rajendra Prasad Parajuli:** conceptualization, methodology, software, data curation, investigation, validation, formal analysis, supervision, visualization, project administration, writing – review and editing. **Pitambar Dhakal:** methodology, writing – review and editing. **Kishor Pandey:** writing – review and editing, resources, supervision, software.

## Disclosure

The final version of the manuscript was reviewed and approved by all the authors prior to submission. All the authors read and approved the final manuscript.

## Ethics Statement

Ethical approval for this study was obtained from the Institutional Review Board (IRB) of the Institute of Science and Technology (IOST), Tribhuvan University (Approval No. 23‐0061).

## Conflicts of Interest

The authors declare no conflicts of interest.

## Transparency Statement

The lead author Kishor Pandey affirms that this manuscript is an honest, accurate, and transparent account of the study being reported; that no important aspects of the study have been omitted; and that any discrepancies from the study as planned (and, if relevant, registered) have been explained.

## Supporting information

Supporting information.

## Data Availability

The data will be available upon request to the corresponding author.

## References

[1] P. R. Torgerson , B. Devleesschauwer , N. Praet , et al., “World Health Organization Estimates of the Global and Regional Disease Burden of 11 Foodborne Parasitic Diseases, 2010: A Data Synthesis,” PLoS Medicine 12 (2015): e1001920. 26633705 10.1371/journal.pmed.1001920 PMC4668834

[2] S. T. Hajare , R. K. Gobena , N. M. Chauhan , and F. Eriso , “Prevalence of Intestinal Parasite Infections and Their Associated Factors Among Food Handlers Working in Selected Catering Establishments From Bule Hora, Ethiopia,” BioMed Research International 2021 (2021): 6669742. 34458370 10.1155/2021/6669742 PMC8397551

[3] G. L. Ooi and K. H. Phua , “Urbanization and Slum Formation,” Journal of Urban Health 84 (2007): 27–34. 10.1007/s11524-007-9167-5 PMC1891640 17387618

[4] A. Gilbert , “Book Review: The Challenge of Slums: Global Report on Human Settlements 2003,” Progress in Human Geography 29 (2005): 118–120.

[5] D. R. Joshi , “Urbanization Trend in Nepal,” Contemporary Research: An Interdisciplinary Academic Journal 6 (2023): 51–62.

[6] United Nations Department of Economic and Social Affairs , World Urbanization Prospects: The 2018 Revision (ST/ESA/SER. A/420) (New York: United Nations, 2019).

[7] Y. Sultana , G. L. Gilbert , B. N. Ahmed , and R. Lee , “Strongyloidiasis in a High Risk Community of Dhaka, Bangladesh,” Transactions of the Royal Society of Tropical Medicine and Hygiene 106 (2012): 756–762. 23084030 10.1016/j.trstmh.2012.08.011

[8] P. J. Hotez , M. Alvarado , M. G. Basáñez , et al., “The Global Burden of Disease Study 2010: Interpretation and Implications for the Neglected Tropical Diseases,” PLoS Neglected Tropical Diseases 8 (2014): e2865. 25058013 10.1371/journal.pntd.0002865 PMC4109880

[9] J. Bethony , S. Brooker , M. Albonico , et al., “Soil‐Transmitted Helminth Infections: Ascariasis, Trichuriasis, and Hookworm,” Lancet 367 (2006): 1521–1532. 16679166 10.1016/S0140-6736(06)68653-4

[10] S. A. Vilwanathan , A. Lakshmy Jayachandran , B. Kandasamy , A. Professor , and U. Student , “Prevalence of Intestinal Parasitic Infections and Predisposing Factors Among Children in Field Practice Area of Tertiary Care Centre in South India,” International Journal of Medical Microbiology and Tropical Diseases 3 (2017): 45.

[11] B. Bhattachan , J. B. Sherchand , S. Tandukar , B. G. Dhoubhadel , L. Gauchan , and G. Rai , “Detection of *Cryptosporidium parvum* and *Cyclospora cayetanensis* Infections Among People Living in a Slum Area in Kathmandu Valley, Nepal,” BMC Research Notes 10 (2017): 1–5. 28882168 10.1186/s13104-017-2779-2 PMC5590164

[12] M. Dudeja , S. Nandy , A. K. Das , S. Alam , and R. Tiwari , “Prevalence of Intestinal Parasites in Slum Areas of Southern Delhi,” International Journal of Microbiology Research 4 (2012): 312–315.

[13] I. Tiwari , P. Gyawali , and J. R. Subedi , “Intestinal Parasites in the Slum‐Dwelling Population in Naya Bazar, Kaski, Nepal,” Janaki Medical College Journal of Medical Science 6 (2018): 29–35.

[14] A. Hosna , A. Nahar , and H. Khanum , “Occurrence of Intestinal Parasites Among the Slum Children,” Biores Commun 4 (2018): 470–475.

[15] H. Khanum , M. Mukutmoni , J. Uddin , and R. Haque , “Diarrheal Carriage Illness With *Trichuris trichiura* Among the Slum Dwelling Children in Dhaka City,” Bioresearch Communications 2 (2016): 254–258.

[16] L. K. Khanal , S. K. Rai , P. R. Khanal , and G. Ghimire , “Status of Intestinal Parasitosis Among Hospital Visiting Patients in Deukhury Valley, Dang, Nepal,” Nepal Medical College Journal 13 (2011): 100–102. 22364091

[17] R. Kunwar , L. Acharya , and S. Karki , “Trends in Prevalence of Soil‐Transmitted Helminth and Major Intestinal Protozoan Infections Among School‐Aged Children in Nepal,” Tropical Medicine & International Health 21 (2016): 703–719. 27097973 10.1111/tmi.12700

[18] S. Tandukar , S. Ansari , N. Adhikari , et al., “Intestinal Parasitosis in School Children of Lalitpur District of Nepal,” BMC Research Notes 6 (2013): 1. 24207086 10.1186/1756-0500-6-449 PMC3829703

[19] B. K. Shrestha , M. Tumbahangphe , J. Shakya , et al., “Prevalence and Related Risk Factors of Intestinal Parasitosis Among Private School‐Going Pupils of Dharan Submetropolitan City, Nepal,” Marchand B. , editor. Journal of Parasitology Research 2021 (2021): 6632469, 10.1155/2021/6632469. 34306741 PMC8285192

[20] N. Thapa , J. R. Subedi , and B. Chhetri , “Prevalence of Intestinal Parasites Among Sarki Ethnic Group of Pala Rural Municipality, Baglung, Nepal,” Annals of Parasitology 67 (2021): 329–336. 34598405 10.17420/ap6702.346

[21] M. Shobha , D. Bithika , and S. Bhavesh , “The Prevalence of Intestinal Parasitic Infections in the Urban Slums of a City in Western India,” Journal of Infection and Public Health 6 (2013): 142–149. 23537828 10.1016/j.jiph.2012.11.004

[22] S. Khadka , S. Sapkota , S. Adhikari , et al., “Intestinal Parasitoses Among Chepang and Musahar Community People of Makwanpur and Nawalparasi Districts of Nepal,” Acta Parasitologica 66 (2021): 146–154. 32829473 10.1007/s11686-020-00269-0

[23] R. B. Adhikari , R. P. Parajuli , M. Maharjan , and T. R. Ghimire , “Prevalence and Risk Factors of Gastrointestinal Parasites in the Chepangs in Nepal,” Annals of Parasitology 67 (2021): 387–405. 34953115 10.17420/ap6703.353

[24] M. Dahal and A. Jha , “Enteroparasites in the People Living in Slum Area of Thapathali, Kathmandu Valley,” EC Microbiology 5 (2016): 542–547.

[25] M. Marasini and C. L. Chidi , “Vulnerability Assessment of Squatter Settlement in Butwal Sub‐Metropolitan City, Nepal,” Third Pole: Journal of Geography Education 20–21 (2021): 47–58.

[26] R. Chongbang , P. Dongol , A. Chakrawarti , and H. Khanal , “Parasitic Infections Among Children of Squatter Community in Dharan Municipality, Sunsari, Nepal,” International Journal of Applied Sciences and Biotechnology 4 (2016): 203–206.

[27] J. Dhanabal , P. P. Selvadoss , and K. Muthuswamy , “Comparative Study of the Prevalence of Intestinal Parasites in Low Socioeconomic Areas From South Chennai, India,” Journal of Parasitology Research 2014 (2014): 630968. 24587897 10.1155/2014/630968 PMC3918716

[28] S. Fallahizadeh , M. H. Feiz‐Haddad , F. Kazemi , and R. Afrisham , “Prevalence of Intestinal Parasitic Infections in Shush County, Southwest of Iran During 2014–2016,” International Journal of Infection 4 (2017), https://www.researchgate.net/publication/317550301_Prevalence_of_Intestinal_Parasitic_Infections_in_Shush_County_Southwest_of_Iran_during_2014_-_2016.

[29] N. A. Novitasari and M. Z. Fatah , “Systematic Review of Risk Factor of Intestinal Parasite Infection,” Media Gizi Kesmas 10 (2021): 165–179.

[30] A. Mahapatra , N. Mohanty , B. K. Behera , S. Dhal , and A. K. Praharaj , “Soil Transmitted Helminth Infections Among School Going Age Children of Slums From Bhubaneswar, Odisha,” Tropical Parasitology 10 (2020): 34–38. 32775290 10.4103/tp.TP_30_19 PMC7365495

[31] A. M. Zajac , G. A. Conboy , S. E. Little , and M. V. Reichard , Veterinary Clinical Parasitology (John Wiley & Sons, 2021).

[32] E. J. L. Soulsby , Helminths, Arthropods and Protozoa of Domesticated Animals (Bailliere Tindall, 10 Greycoat Place, 1982), https://www.cabidigitallibrary.org/doi/full/10.5555/19832217454.

[33] W. J. Foreyt , Veterinary Parasitology Reference Manual (John Wiley & Sons, 2013).

[34] P. Dhakal , H. P. Sharma , R. Shah , P. J. Thapa , and C. P. Pokheral , “Copromicroscopic Study of Gastrointestinal Parasites in Captive Mammals at Central Zoo, Lalitpur, Nepal,” Veterinary Medicine and Science 9 (2023): 457–464. 36495198 10.1002/vms3.1039 PMC9857001

[35] S. C. Parija , “Textbook of Medical Parasitology, Protozoology & Helminthology,” Revista do Instituto de Medicina Tropical de Sao Paulo 50 (2008): 282.

[36] A. M. Zajac , G. A. Conboy , E. C. Greiner , S. A. Smith , and K. F. Snowden , “Fecal Examination for the Diagnosis of Parasitism,” Veterinary Clinical Parasitology 8 (2012): 3–169.

[37] C. F. Ignacio , M. E. C. da Silva , N. B. Handam , et al., “Socioenvironmental Conditions and Intestinal Parasitic Infections in Brazilian Urban Slums: A Cross‐Sectional Study,” Revista do Instituto de Medicina Tropical de São Paulo 59 (2017): 1–10. 10.1590/S1678-9946201759056 PMC5553943 28793024

[38] T. Kuete , F. L. S. Yemeli , E. E. Mvoa , T. Nkoa , R. M. Somo , and A. S. Ekobo , “Prevalence and Risk Factors of Intestinal Helminth and Protozoa Infections in an Urban Setting of Cameroon: The Case of Douala,” American Journal of Epidemiology and Infectious Disease 3 (2015): 36–44.

[39] F. F. Gil , H. G. N. O. Busatti , V. L. Cruz , J. F. G. Santos , and M. A. Gomes , “High Prevalence of Enteroparasitosis in Urban Slums of Belo Horizonte‐Brazil. Presence of Enteroparasites as a Risk Factor in the Family Group,” Pathogens and Global Health 107 (2013): 320–324. 24091002 10.1179/2047773213Y.0000000107 PMC4001612

[40] S. Jamali , M. S. Khan , S. Azmi , A. Ahmad , and U. Pradesh , “Intestinal Parasitic Infestation in an Urban Slum of Lucknow City,” International Journal of Medical Science and Education 7 (2020): 28–34.

[41] M. Mukutmoni and H. Khanum , “Prevalence and Risk Factors of Intestinal Helminthiasis Among the Children of Begun Bari Slum, Tejgaon, Dhaka,” Bangladesh Journal of Zoology 45 (2018): 123–129.

[42] A. J. Zeleke , A. G. Bayih , S. Afework , and J. S. Gilleard , “Treatment Efficacy and Re‐Infection Rates of Soil‐Transmitted Helminths Following Mebendazole Treatment in Schoolchildren, Northwest Ethiopia,” Tropical Medicine and Health 48 (2020): 90. 33292853 10.1186/s41182-020-00282-z PMC7659054

[43] R. P. Parajuli , M. Umezaki , and C. Watanabe , “Behavioral and Nutritional Factors and Geohelminth Infection Among Two Ethnic Groups in the Terai Region, Nepal,” American Journal of Human Biology 21 (2009): 98–104. 18802944 10.1002/ajhb.20825

[44] P. J. Hotez , S. Brooker , J. M. Bethony , M. E. Bottazzi , A. Loukas , and S. Xiao , “Hookworm Infection,” New England Journal of Medicine 351 (2004): 799–807. 15317893 10.1056/NEJMra032492

[45] I. D. Amoah , G. Singh , T. A. Stenström , and P. Reddy , “Detection and Quantification of Soil‐Transmitted Helminths in Environmental Samples: A Review of Current State‐of‐the‐Art and Future Perspectives,” Acta Tropica 169 (2017): 187–201. 28214519 10.1016/j.actatropica.2017.02.014

