# Supplementary material for: Intestinal Parasitic Infections and Associated Behavioral Risk Factors in a Squatter Community in Butwal, Nepal: A Pilot Study
Source: Health Sci Rep. 2025 Feb 16;8(2):e70473. doi: 10.1002/hsr2.70473 (PMC11830998; doi:10.1002/hsr2.70473)

Supplementary Figure 1. Bar graph representing the behavioral, lifestyle characteristics of the study participants.


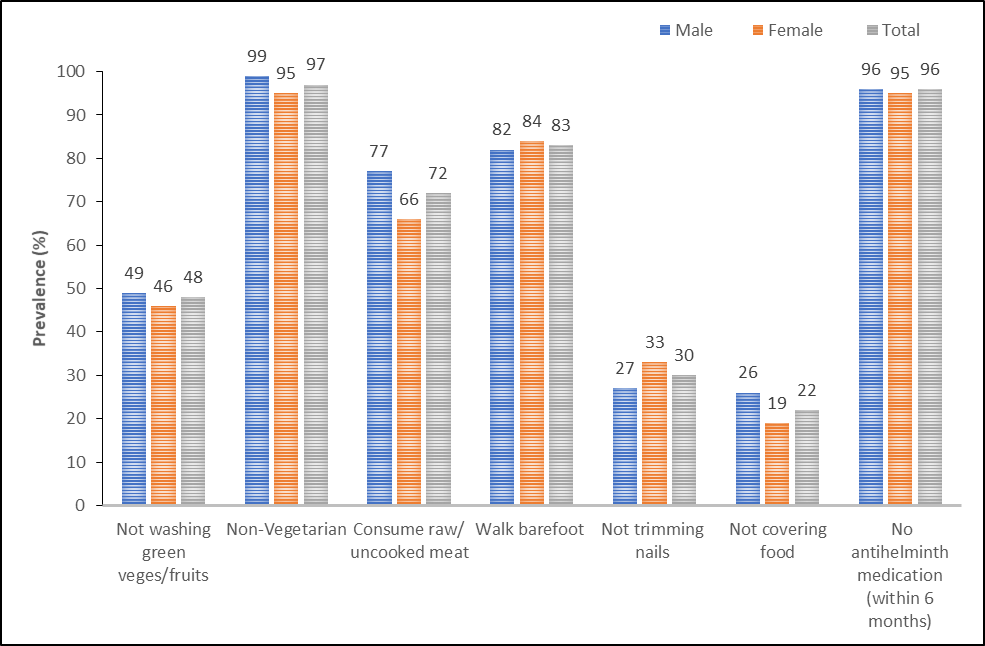

Supplement: Supplementary file 1 — Supporting information. [file HSR2-8-e70473-s001.docx]
